# Supplementary material for: Perceived Social Support Increases Creativity: Experimental Evidence
Source: Int J Environ Res Public Health. 2022 Sep 19;19(18):11841. doi: 10.3390/ijerph191811841 (PMC9517368; doi:10.3390/ijerph191811841)
Supplement: Supplementary file 1 [file ijerph-19-11841-s001.zip › ijerph-1894704-supplementary.pdf]

Table S1

Mean and Standard Deviation of the Log-transformed Creativity Measures in the Experimental and Control Groups

		Experimental (n = 69)		Control (n = 66)	
No.	Variable	Mean	*SD*	Mean	*SD*
1	Fluency	1.13	0.31	1.00	0.30
2	Flexibility	1.00	0.29	0.87	0.27
3	Originality	1.31	0.35	1.11	0.35
4	Self-rated creativity	0.57	0.05	0.54	0.05

Note. N = 135. All differences between the means in both groups are statistically significant at .05 level.
